# Eligibility for Bevacizumab as an Independent Prognostic Factor for Patients with Advanced Non-Squamous Non-Small Cell Lung Cancer: A Retrospective Cohort Study

**DOI:** 10.1371/journal.pone.0059700

**Published:** 2013-03-26

**Authors:** Yusuke Takagi, Akira Toriihara, Yoshiro Nakahara, Makiko Yomota, Yusuke Okuma, Yukio Hosomi, Masahiko Shibuya, Tatsuru Okamura

**Affiliations:** 1 Department of Thoracic Oncology and Respiratory Medicine, Tokyo Metropolitan Cancer and Infectious Diseases Center Komagome Hospital, Tokyo, Japan; 2 Department of Diagnostic Radiology and Oncology, Tokyo Medical and Dental University, Tokyo, Japan; University of Porto, Portugal

## Abstract

**Background:**

Bevacizumab requires some unique eligibility criteria, such as absence of hemoptysis and major blood vessel invasion by the tumor. The prognostic impact of these bevacizumab-specific criteria has not been evaluated.

**Methods:**

Patients with stage IIIB/IV, non-squamous non-small cell lung cancer who started chemotherapy before the approval of bevacizumab were reviewed. Patients with impaired organ function, poor performance status or untreated/symptomatic brain metastasis were excluded before the evaluation of bevacizumab eligibility. We compared overall survival and time to treatment failure among patients who were eligible (Group A) or ineligible (Group B) to receive bevacizumab.

**Results:**

Among 283 patients with stage IIIB/IV non-squamous non-small cell lung cancer, eligibility for bevacizumab was evaluated in 154 patients. Fifty-seven patients were considered ineligible (Group B) based on one or more of a history of hemoptysis (*n* = 20), major blood vessel invasion (*n* = 43) and cardiovascular disease (*n* = 8). The remaining 97 patients were classified into Group A. Overall survival was significantly better in Group A (median, 14.6 months) than in Group B (median, 7.1 months; *p*<0.0001). Time to treatment failure was also significantly longer in Group A (median, 6.9 months) than in Group B (median, 3.0 months; *p*<0.0001). Adjusted hazard ratios of bevacizumab eligibility for overall survival and time to treatment failure were 0.48 and 0.38 (95% confidence intervals, 0.33–0.70 and 0.25–0.58), respectively.

**Conclusion:**

Eligibility for bevacizumab itself represents a powerful prognostic factor for patients with non-squamous non-small cell lung cancer. The proportion of patients who underwent first-line chemotherapy without disease progression or unacceptable toxicity can also be biased by bevacizumab eligibility. Selection bias can be large in clinical trials of bevacizumab, so findings from such trials should be interpreted with extreme caution.

## Introduction

Eligibility is often narrowed in clinical trials of targeted drugs because of specific adverse effects [1]. This is intended to exclude patients who might be at high risk of developing severe adverse events and to maximize the overall efficacy of the drug of interest. As a result, modified eligibility criteria can affect endpoints such as overall survival (OS) independently of the actual effect of an investigational drug.

Bevacizumab (BV), an anti-vascular endothelial growth factor antibody, requires modified eligibility criteria such as absence of hemoptysis and major blood vessel invasion (MVI) in clinical trials [2]–[4]. Some studies have indicated that patients who meet the eligibility criteria for BV are in the minority in the real world [5], but the impacts of these criteria on survival and treatment efficacy have not been evaluated. Understanding the potential selection bias derived from BV-specific eligibility criteria is important for clinicians, so that the results of key clinical trials can be interpreted appropriately.

We investigated whether the eligibility criteria characteristically applied for BV lead to selection bias. This retrospective cohort study examined the relationship between eligibility for BV and prognosis among patients with non-squamous non-small cell lung cancer (NSCLC), by enrolling patients who started chemotherapy before BV gained approval for use in Japan.

## Methods

### Ethics statement

This study was approved by the institutional review board of Tokyo Metropolitan Cancer and Infectious Diseases Center Komagome Hospital (Tokyo, Japan). We used routinely collected data and anonymized data for all analyses, and individual patient consent was not required. The waiver of need for written informed consent was also approved by the institutional review board of Tokyo Metropolitan Cancer and Infectious Diseases Center.

### Data source

Patients were identified from the database at Tokyo Metropolitan Cancer and Infectious Diseases Center and included those who had undergone systemic chemotherapy for the treatment of lung cancer at the Department of Thoracic Oncology and Respiratory Medicine.

### Study participants

Patients with stage IIIB/IV non-squamous NSCLC who started chemotherapy between 2005 and 2009 were reviewed. After receiving approval as a therapeutic drug for treating lung cancer in Japan in November 2009, BV was first applied to treat lung cancer at our institution in 2010. Lung cancer was staged according to the 7th edition of the TNM Classification of Malignant Tumors [6] by the International Union Against Cancer (UICC). Tumors with mixed histological subtypes of NSCLC were categorized into a subtype according to the predominant component.

We excluded patients with indications for combined chemoradiotherapy, Eastern Cooperative Oncology Group (ECOG) performance status (PS) 3 or 4, untreated or symptomatic brain metastasis or impaired bone marrow, hepatic or renal function at the start of chemotherapy, because these patients are excluded from most clinical trials of first-line chemotherapy for lung cancer [7], [8]. Patients with PS 2 were included because the AVAPERL study did not exclude these patients [4]. We included patients without information about PS in survival analyses, because patients with PS 3/4 rarely start chemotherapy without this being specifically mentioned in the medical records.

### Evaluation of eligibility for bevacizumab

Patients were considered ineligible for BV if they had one or more of a history of hemoptysis, MVI by the tumor and clinically significant cardiovascular disease (CVD).

A history of hemoptysis was defined as an episode of hemoptysis within 3 months prior to starting chemotherapy. Since investigating the volume of expectorated blood is difficult, all episodes of hemoptysis were included, irrespective of severity. A radiologist who was blinded to clinical outcomes evaluated MVI. Major blood vessels included the aorta, superior vena cava, inferior vena cava, main pulmonary arteries and main branches of the pulmonary arteries and pulmonary veins within the pericardial sac. Invasion was defined as contact of >180°with these vessels [9] or an irregular bump in the vessels. We defined CVD as chronic congestive heart failure, ischemic heart disease or vascular disease (including thrombotic events) requiring medication.

If any one of these three elements was identified, the patient was considered ineligible for BV (Group B). When all elements were negative, the patient was assigned to Group A. If data about any elements were unobtainable and all other elements were negative, eligibility could not be determined and the patient was excluded from survival analysis.

### Survival outcomes

The primary endpoint was OS, defined as the number of months between starting the first chemotherapy regimen until the date of death. Patients alive at the end of follow-up were censored, except for those with disease progression who were unwilling to undergo further anticancer therapy. To diminish the influence of informative censoring, the last follow-up was regarded as an event in these patients (for example, lost to follow-up after transfer to hospice).

### Time to treatment failure

We defined the time to treatment failure (TTF) of patients who underwent first-line chemotherapy containing platinum as the number of months that elapsed between the initiation of chemotherapy until the date of starting any type of subsequent therapy, request from the patient to terminate anticancer therapy, or death. Follow-up duration was defined as the time elapsed from initiating chemotherapy until the date of death or last follow-up. Subsequent therapy included chemotherapy, radiotherapy and tumor resection. Patients who remained alive without subsequent therapy at the end of follow-up were censored.

### Control variables

We collected information about age, sex, histological type of NSCLC, stage of lung cancer, laboratory findings and PS at the time of chemotherapy initiation from medical records. Tumor histology categories were defined as adenocarcinoma, NSCLC not otherwise specified (NOS), large cell neuroendocrine carcinoma (LCNEC) and others. We also surveyed the first chemotherapeutic regimen and subsequent therapies.

Sex, disease stage (IIIB or IV), PS (0, 1 or 2) and platinum as first-line chemotherapy were included as explanatory variables, because these factors are known to affect the survival of patients with NSCLC [10], [11]. Whether platinum is beneficial for elderly patients remains unclear [12], so we also included age (<70 vs. ≥70 years) among the variables. A history of hemoptysis, MVI and CVD were also included among the variables. Number of metastatic sites, weight loss and laboratory markers were not included because the relevance of the prognostic impact of these markers has not appeared constant through a number of studies [13]–[16]. Patients with insufficient information about any variable were excluded from multivariate analysis. Since epidermal growth factor receptor (EGFR) mutation status was known for only a small number of patients, EGFR mutations were not included as variables in this analysis.

### Study size

We designed the study assuming that median OS of the BV-eligible cohort would be 1.5 years with a hazard ratio of 0.5, based on previous findings [3], [17]. To detect a difference in OS with 90% power for a two-sided significance value of 0.05, 121 patients were required. Our database accumulates 100–120 patients with lung cancer annually, with about 50% having presumed advanced non-squamous NSCLC, and almost half meeting the eligibility criteria of ordinary clinical trials. We therefore analyzed data from 2005 to 2009 to accumulate sufficient subjects.

### Statistical methods

Differences in characteristics except for age between Groups A and B were evaluated using the χ^2^ test. Differences in age were compared using Student's t-test. Survival and TTF were estimated using the Kaplan-Meier method and the log-rank test was used for inter-group comparisons. We also examined the prognostic impact of the variables described above on OS and TTF using Cox proportional hazards modeling. All tests were two-sided with a significance level of 0.05. All data were analyzed using JMP version 9.0 software (SAS Institute, Cary, NC, USA).

## Results

### Patient characteristics

**Figure 1:**
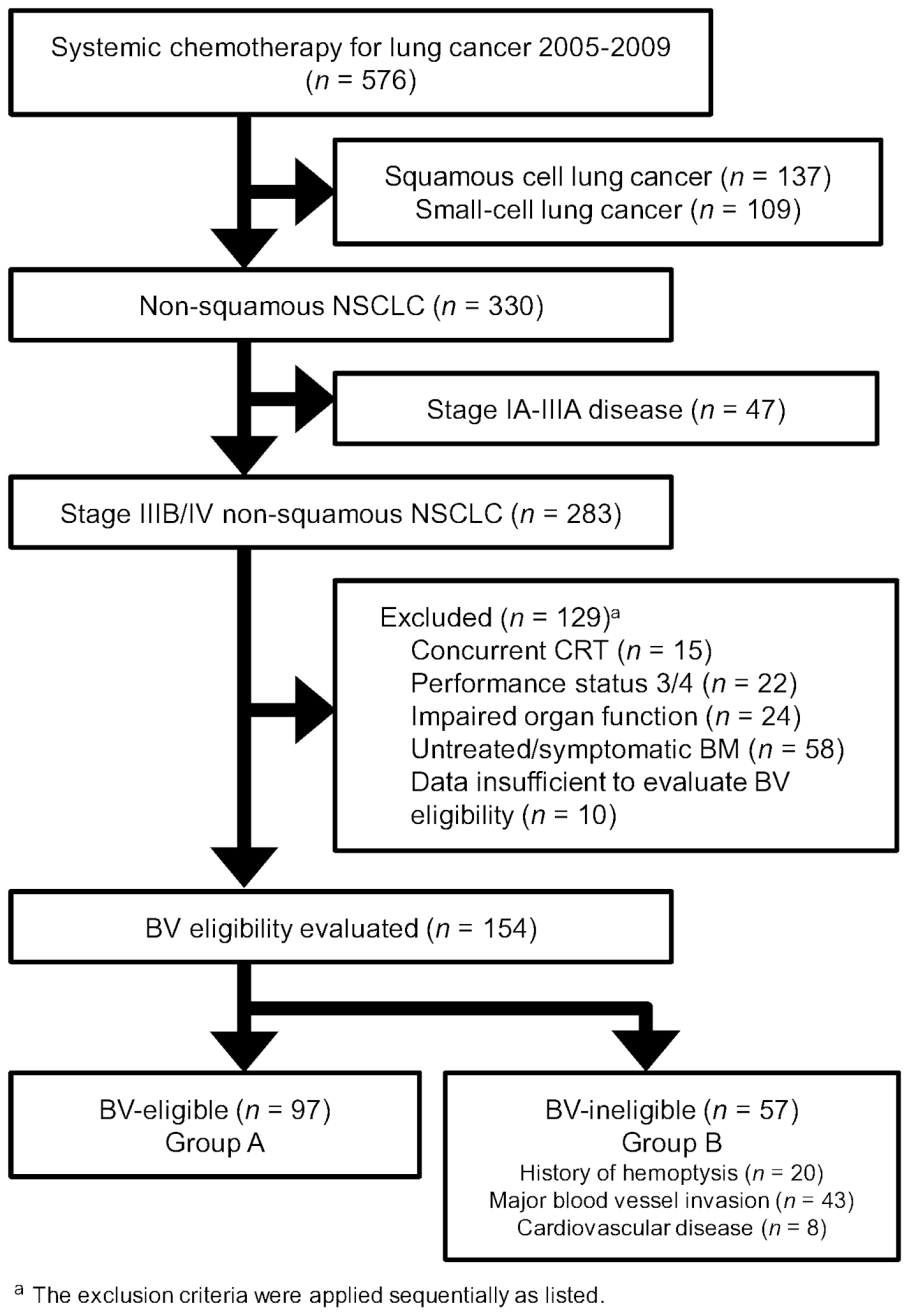
Flow chart of patients through the study. BM, brain metastasis; BV, bevacizumab; CRT, chemoradiotherapy; NSCLC, non-small cell lung cancer.

Among 576 patients with lung cancer treated at our hospital between 2005 and 2009, a total of 283 had stage IIIB/IV non-squamous NSCLC. BV was not administered to any of these patients throughout the course of treatment. After excluding 129 patients showing indications for concurrent chemoradiotherapy, PS 3 or 4, impaired organ function, untreated or symptomatic brain metastasis or insufficient information, we evaluated the eligibility of the remaining 154 patients for BV. Fifty-seven patients were considered ineligible for BV (Group B), based on one or more of a history of hemoptysis (*n* = 20), MVI (*n* = 43) or CVD (*n* = 8). The remaining 97 patients were assigned to Group A (Figure 1).

The median age of Groups A and B combined (*n* = 154) was 67 years (range, 41–84 years), and 54 patients (35%) were female. Most patients had adenocarcinoma (84%) and stage IV disease (92%). The mutation status of EGFR was determined in 39 (25%) patients. The proportions of patients with EGFR mutations among those for whom EGFR status was determined did not differ significantly between groups, at 10 of 30 (33%) in Group A and 4 of 9 (44%) in Group B.

Baseline characteristics of both groups were similar, although the proportion of patients with PS 1 was rather higher in Group B (Table 1). Proportions of PS 2 patients among Groups A and B were almost equal. Platinum-based chemotherapy was administered to 77 patients (79%) in Group A and 44 patients (77%) in Group B, showing no significant difference between groups.

**Table 1:** Baseline characteristics.

Category	Subcategory	Group A, *n* (%)	Group B, *n* (%)	*p*
Total		97	57	
Median age (range), y		67 (41–83)	70 (50–84)	0.2
Sex	Female	36 (37)	18 (32)	0.5
	Male	61 (63)	39 (68)	
Disease stage	IIIB	8 (8)	4 (7)	0.8
	IV	89 (92)	53 (93)	
Histological type	Adenocarcinoma	84 (87)	45 (79)	0.3
	NSCLC, NOS	8 (8)	5 (9)	
	LCNEC	2 (2)	5 (9)	
	Other	3 (3) b	2 (4) c	
EGFR mutation	Negative	20 (21)	5 (9)	0.08
	Positive	10 (10)	4 (7)	
	Unknown	67 (69)	48 (84)	
PS a	0	38 (39)	13 (23)	0.04
	1	40 (41)	34 (60)	
	2	16 (16)	10 (18)	

a PS of three patients in Group A was unknown.

b Three adenosquamous carcinomas.

c Pleomorphic carcinoma and undifferentiated carcinoma.

EGFR, epidermal growth factor receptor; LCNEC, large cell neuroendocrine carcinoma;

NOS, not otherwise specified; NSCLC, non-small cell lung cancer;

PS, performance status.

### Overall survival

**Figure 2:**
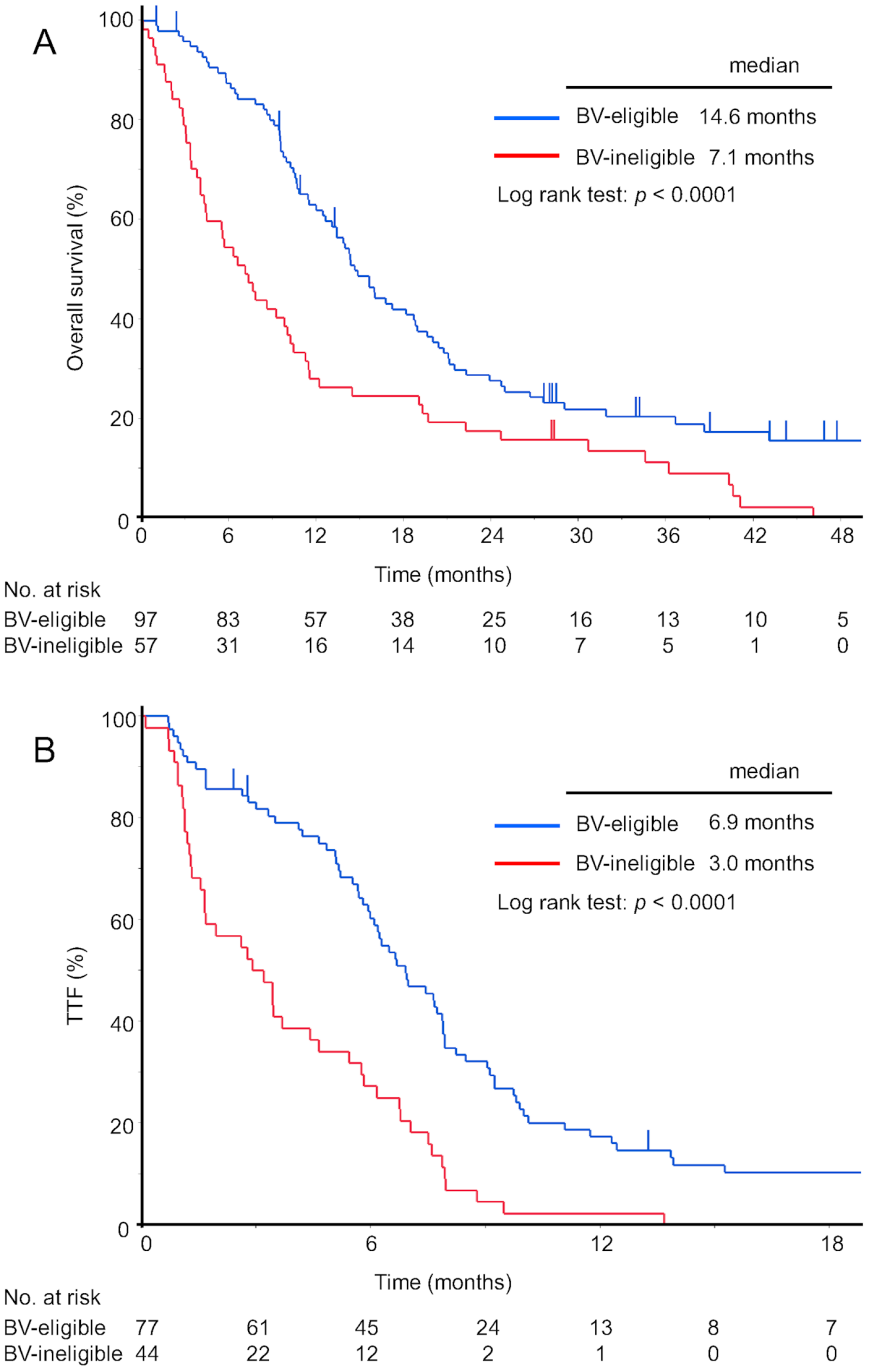
Overall survival and time to treatment failure for patients eligible compared with ineligible for bevacizumab. Kaplan-Meier curves for overall survival (A) and time to treatment failure (B). BV, bevacizumab; TTF, time to treatment failure.

Median OS was significantly better in Group A (14.6 months) than in Group B (7.1 months; *p*<0.0001) (Figure 2). The crude hazard ratio of BV eligibility for OS was 0.50 (95% confidence interval (CI), 0.36–0.72). One-year survival rates for Groups A and B were 62% and 28%, respectively. Differences in OS between groups remained even after censoring patients with disease progression and those who declined further therapy at the end of follow-up (median OS, 16.8 and 7.8 months, respectively; *p* = 0.0001). Median OS was significantly longer in Group A than in Group B among 121 patients who had received first-line chemotherapy with platinum (18.8 vs. 9.2 months; *p* = 0.0006).

**Figure 3:**
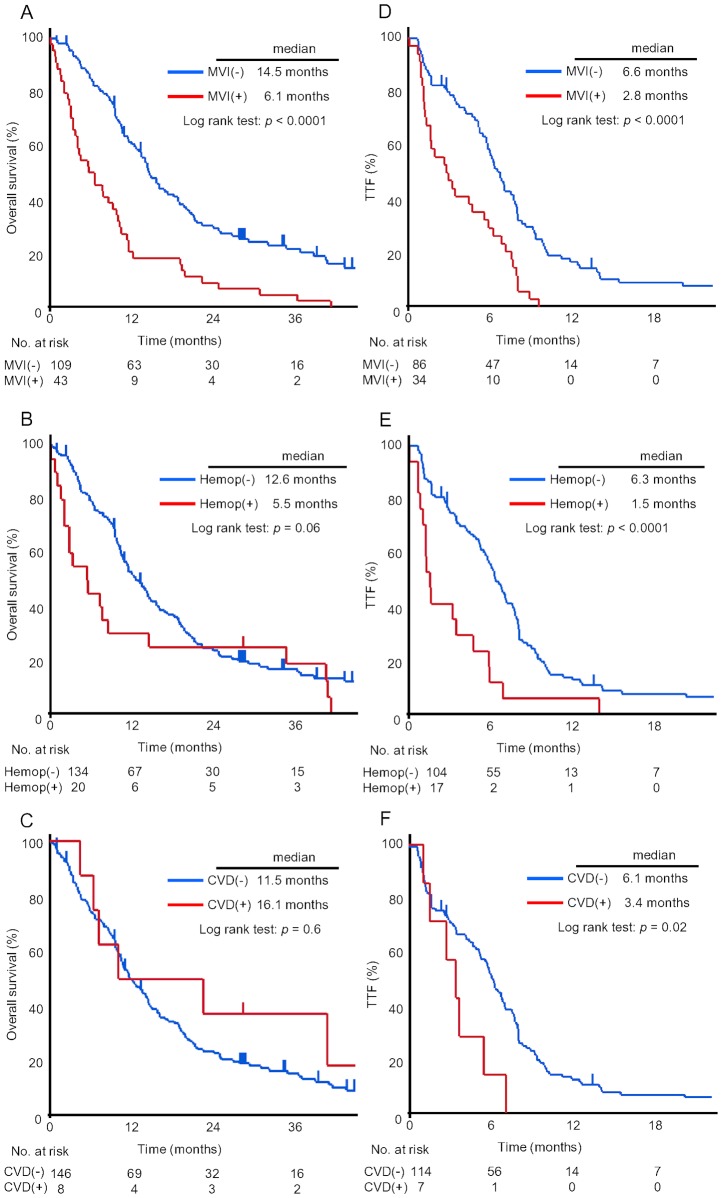
Overall survival and time to treatment failure by each condition defining the eligibility for bevacizumab. Kaplan-Meier curves for overall survival by MVI (A), history of hemoptysis (B) and CVD (C) and time to treatment failure by MVI (D), history of hemoptysis (E) and CVD (F). CVD, cardiovascular disease; Hemop, history of hemoptysis; MVI, major blood vessel invasion; TTF, time to treatment failure.

Multivariate analysis indicated PS 2, use of platinum, history of hemoptysis and MVI as significant prognostic factors (Table 2). The adjusted hazard ratio of BV eligibility for other variables was 0.48 (95%CI, 0.33–0.70, p = 0.0001), indicating that BV eligibility itself represents an independent prognostic factor for patients with advanced non-squamous NSCLC. Prognostic impact of BV eligibility consists of MVI and history of hemoptysis, with CVD exerting no influence (Figure 3).

**Table 2:** Cox proportional hazards models for overall survival.

Variable	Value	HR	95% CI	*p*
Use of platinum	Yes	1	Reference	
	No	2.05	1.20 to 3.41	0.009
Performance status	0	1	Reference	
	1	0.95	0.63 to 1.45	0.8
	2	2.16	1.21 to 3.79	0.01
History of hemoptysis	No	1	Reference	
	Yes	1.89	1.07 to 3.20	0.03
Major blood vessel invasion	No	1	Reference	
	Yes	2.59	1.70 to 3.91	<0.0001

CI, confidence interval; HR, hazard ratio.

Among 61 patients younger than 65 years, OS was also significantly better in Group A (14.8 months) than in Group B (7.8 months; *p* = 0.003). Median OS was 26.1 months for patients with mutated EGFR and 16.0 months for patients with wild-type EGFR. This difference was not significant, because the EGFR status of most patients was unknown. Median OS was 16.0 months in Group A and 9.2 months in Group B, respectively, among the 25 patients with wild-type EGFR, and 22.1 months and 35.4 months, respectively, in the 14 patients with mutated EGFR. These preliminary findings did not reflect a significant difference, probably due to the small size of each subgroup.

### Time to treatment failure

The median duration of follow-up among the 121 patients who underwent platinum-based first-line chemotherapy was 12.6 months. TTF was significantly better in Group A (6.9 months) than in Group B (3.0 months; *p*<0.0001) (Figure 2). The crude hazard ratio of BV eligibility to TTF was 0.39 (95%CI, 0.25–0.58). The proportions of Groups A and B with a TTF >3 months were 82% and 50%, respectively. Multivariate analysis indicated that stage IV disease, history of hemoptysis, MVI and CVD are associated with shorter TTF (Table 3). The adjusted hazard ratio of BV eligibility to other baseline variables was 0.38 (95%CI, 0.25–0.58; *p*<0.0001). More patients underwent consequent chemotherapy in Group A (74%) than in Group B (50%, *p* = 0.008), and the median number of chemotherapy regimens was three in group A and two in group B (*p* = 0.003).

**Table 3:** Cox proportional hazards models for time to treatment failure.

Variable	Value	HR	95% CI	*p*
Disease stage	IIIB	1	Reference	
	IV	2.32	1.19 to 5.11	0.01
History of hemoptysis	No	1	Reference	
	Yes	2.84	1.50 to 5.13	0.002
Major blood vessel invasion	No	1	Reference	
	Yes	2.44	1.52 to 3.86	0.0003
Cardiovascular disease	No	1	Reference	
	Yes	3.04	1.16 to 6.99	0.03

CI, confidence interval; HR, hazard ratio.

## Discussion

This study showed that eligibility for BV itself represents a significant prognostic factor for patients with non-squamous NSCLC. Patients unsuitable for participating in ordinary clinical trials were excluded ahead of the evaluation of BV eligibility, therefore our findings suggest that eligibility for BV lead to substantial selection bias for OS in clinical trials of NSCLC. Multivariate analysis also revealed that two of the three factors that define eligibility for BV strongly impacted OS. Although more patients in Group B tended to have PS 1, OS did not differ significantly between patients with PS 0 and 1. The difference in baseline PS between groups thus had minimal effect on the present results.

We also found that BV eligibility is associated with a longer TTF. Patients with a TTF longer than a certain time period indicate individuals who underwent chemotherapy without disease progression or unacceptable toxicity. When maintenance therapy was preplanned, longer TTF results in higher proportions of patients who actually receive maintenance therapy. Progression-free survival (PFS) is generally favorable for evaluating the impact on treatment efficacy, since TTF is affected not only by efficacy, but also by toxic effects. However, we did not use PFS in this retrospective study because intervals between radiographic evaluations varied considerably among patients, which would compromise the accuracy of the results [18]. Our results support the notion that BV eligibility confers a positive impact upon chemotherapeutic outcomes, but this speculation requires validation.

As far as we can determine, this is the first study to directly compare the prognosis of patients with advanced non-squamous NSCLC in terms of eligibility for BV. Patients participating in clinical trials generally have relatively better prognosis because of strict criteria that exclude patients showing established risk factors for early death, but the prognostic impact of BV-specific eligibility criteria among these selected patients was unknown. Our findings that BV-specific eligibility leads to better prognosis may explain why the OS of the control arm (carboplatin-paclitaxel) in ECOG 4599 [9] was relatively longer than that in other contemporary trials [19]. Similarly, a randomized phase II trial of the same design as ECOG 4599 in Japan found that median OS of the control arm was 23.4 months [17], considerably longer than the median 13.3 months for the carboplatin-paclitaxel arm in a Japanese phase III trial [20]. The concern about selection bias derived from BV eligibility has also been raised in studies of patients with colorectal cancer [21]. These and the present findings indicate that imposing eligibility criteria with the intention of avoiding severe adverse events also helps to improve survival.

The key limitation of this retrospective study is that the findings were derived from data generated at a single institution. We attempted to minimize bias by establishing objective criteria to define eligibility for BV based on large, completed phase III trials [4], [17], and having a blinded radiologist assess MVI. Taking the large magnitude of difference in OS and TTF between the two groups into account, the probability of this study is considerable. Second, some of the eligibility criteria for BV applied herein are no longer being applied in some ongoing phase III studies [22]. However, whether patients who were ineligible for BV based on previous pervasive criteria [1] and then eligible under the new criteria will necessarily enter into current trials is questionable. Although the exclusion criteria for BV were relaxed, resolving the prognostic impact of eligibility for BV might take some time. Third, we did not fully evaluate the impact of EGFR mutations in this study. Mutated EGFR is associated with longer OS in the era of targeted therapies [23], [24]. The proportions of patients with determined EGFR mutations were similar between groups, but the actual relationship between BV eligibility and EGFR mutation status has yet to be clarified.

ECOG 4599 demonstrated a significant survival advantage by adding BV to carboplatin-paclitaxel [2]. However, whether adding BV to cisplatin-based chemotherapy also prolongs survival remains controversial [3]. A recent large observational study of over 4,000 patients found no significant benefit of adding BV to carboplatin-paclitaxel in patients ≥65 years old [25]. Suitable patient subgroups or concomitant chemotherapies that will maximize the benefit of BV should be identified, since addition of BV is associated with a higher incidence of treatment-related death [26]. However, few studies are designed for randomization based on the presence or absence of BV. Clinicians must therefore design therapeutic strategies by extrapolating the outcomes of trials conducted under various settings. This study showed that selection bias can be substantial in clinical trials of BV. As a result, relatively better OS and success rate of induction chemotherapy observed in trials not based on randomization in terms of administration of BV are not sufficient grounds for using BV with cisplatin-based standard chemotherapy.

In conclusion, eligibility for BV represents a powerful prognostic factor for patients with non-squamous NSCLC, regardless of BV use. The impact of selection bias should be carefully considered when interpreting the results of trials with modified eligibility criteria. Further study is warranted to validate our findings.
